# HbSnRK2.6 Functions in ABA-Regulated Cold Stress Response by Promoting HbICE2 Transcriptional Activity in *Hevea brasiliensis*

**DOI:** 10.3390/ijms222312707

**Published:** 2021-11-24

**Authors:** Xue Wang, Wen-Cheng Liu, Xue-Wei Zeng, Sa Yan, Yi-Min Qiu, Jin-Bo Wang, Xi Huang, Hong-Mei Yuan

**Affiliations:** 1 Hainan Key Laboratory for Sustainable Utilization of Tropical Bioresources, College of Tropical Crops, Hainan University, Haikou 570228, China; wangxuer1111@163.com (X.W.); s1769859236@yahoo.com (X.-W.Z.); yansa98411@sina.com (S.Y.); hy0206070@hainmc.edu.cn (Y.-M.Q.); jinbo20161227@163.com (J.-B.W.); xihuang@hainu.edu.cn (X.H.); 2 State Key Laboratory of Crop Stress Adaptation and Improvement, School of Life Sciences, Henan University, Kaifeng 475004, China; liuwencheng@henu.edu.cn

**Keywords:** cold stress tolerance, ABA, *Hevea brasiliensis*

## Abstract

Low temperature remarkably limits rubber tree (*Hevea brasiliensis* Muell. Arg.) growth, latex production, and geographical distribution, but the underlying mechanisms of *Hevea brasiliensis* cold stress response remain elusive. Here, we identified HbSnRK2.6 as a key component in ABA signaling functions in phytohormone abscisic acid (ABA)-regulated cold stress response in *Hevea brasiliensis*. Exogenous application of ABA enhances *Hevea brasiliensis* cold tolerance. Cold-regulated (COR) genes in the CBF pathway are upregulated by ABA. Transcript levels of all five HbSnRK2.6 members are significantly induced by cold, while *HbSnRK2.6A*, *HbSnRK2.6B*, and *HbSnRK2.6C* can be further activated by ABA under cold conditions. Additionally, HbSnRK2.6s are localized in the cytoplasm and nucleus, and can physically interact with HbICE2, a crucial positive regulator in the cold signaling pathway. Overexpression of *HbSnRK2.6A* or *HbSnRK2.6B* in *Arabidopsis* extensively enhances plant responses to ABA and expression of COR genes, leading to increased cold stress tolerance. Furthermore, HbSnRK2.6A and HbSnRK2.6B can promote transcriptional activity of HbICE2, thus, increasing the expression of *HbCBF1*. Taken together, we demonstrate that HbSnRK2.6s are involved in ABA-regulated cold stress response in *Hevea brasiliensis* by regulating transcriptional activity of HbICE2.

## 1. Introduction

The rubber tree (*Hevea brasiliensis* Muell. Arg.) is an economically important tropical tree species, and the latex in its laticifers is the exclusive commercial source of natural rubber, an indispensable industrial raw material [1]. As a representative tropical rainforest tree species, the growth and development of *Hevea brasiliensis* are promoted under warm temperatures while they are suppressed by low temperatures, which restrict its geographical distribution and decrease latex production [2,3,4]. Low temperatures can cause suspension of latex production for 1–3 months per year. In addition, cold stress threatens the survival of young rubber trees, especially in the marginal northern tropics [5,6]. Therefore, improvement of cold tolerance is one of the major tasks of *Hevea brasiliensis* breeding programs. However, there have been few studies on the cold tolerance of *Hevea brasiliensis*, and the key regulators and underlying mechanisms of *Hevea brasiliensis* cold response are largely unknown. Thus, identification of potential genes that function in cold response and illustration of the cold tolerance mechanisms are essential for the development of novel genetic resources for breeding of cold-tolerant *Hevea brasiliensis* varieties.

Plants are sessile organisms that have evolved sophisticated mechanisms for perceiving, transmitting, and responding to cold stress signals by regulating their molecular, biochemical, and physiological processes, thus, enabling them to survive under adverse environmental conditions [7,8,9]. Over the past two decades, enormous achievements have been made in identifying the crucial components implicated in plant cold signaling pathways and deciphering their regulatory mechanisms using the model plant *Arabidopsis* [8,10,11]. The best understood cold regulatory pathway, the inducer of CBF expression (ICE)-C-repeat binding factor/DRE binding factor1 (CBF/DREB1) transcriptional cascade, plays a critical role in cold acclimation in Arabidopsis [9,12]. The CBFs/DREBs in this pathway are rapidly activated by cold, which can bind to the promoters of the cold-regulated (COR) genes to upregulate their expression, thus, increasing plant cold tolerance [13]. Several transcription factors activating or suppressing the expression of cold-induced CBF have been identified. For instance, ICE1, brassinazole-resistant 1 (BZR1), and calmodulin-binding transcription activator 3 (CAMTA3) and CESTA are positive regulators of CBF genes [14,15,16,17]. By contrast, MYB15, ethylene insensitive 3 (EIN3) and phytochrome-interacting factor 3/4/7 (PIF3/4/7) directly inhibit cold-induced CBF expression [18,19,20,21]. ICE1, the best characterized upstream component of CBF, acts as a master regulator of CBF, which binds to the promoter of CBF and activates its transcription [14]. Recent studies have uncovered that ICE1 can be degraded by the E3 ligase high expression of osmotically responsive gene 1 (HOS1)-mediated ubiquitination, while it can be stabilized by the SAP and Miz1 (SIZ1)-mediated sumoylation [22,23]. In addition, phosphorylation of ICE1 is important for its function in plant cold tolerance. It has been reported that ICE1 can be phosphorylated by several protein kinases, including open stomata 1 (OST1)/SNF1-related protein kinase 2.6 (SnRK2.6), mitogen-activated protein kinase 3/6 (MPK3/6), and brassinosteroid-insensitive 2 (BIN2) in *Arabidopsis*, and OsMAPK3 in rice [21,24,25,26].

Recent reports have also documented that plant hormones play important roles in the regulation of coordinating ICE/CBF cold regulatory pathway to modulate plant cold tolerance [27,28]. For example, ethylene has been shown to decrease plant freezing tolerance through inhibiting the expression of CBFs and type-A ARR genes in *Arabidopsis* [19]. In addition, exogenous application of 24-epibrassinolide (EBR), analogs of brassinosteroids, reduced the expression levels of the ICE1 gene under low-temperature stress (9 °C) in tomato seedlings [29]. Jasmonic acid (JA) has been reported to positively regulate freezing tolerance by regulating the transcriptional activity of ICE1 through its interaction with JA signaling repressor JAZ1/4 in *Arabidopsis* [30]. As the most important stress hormone in plants, abscisic aid (ABA) also plays roles in plant response to low temperature. For example, an early report documented that exogenous application of ABA enhanced *Arabidopsis* tolerance to cold stress [31]. However, whether and how ABA regulates the cold stress response of rubber trees remain elusive.

SnRK2.6, a serine/threonine protein kinase, is a key positive regulator of ABA signaling in plants [32]. SnRK2.6, along with ABA receptors PYR/PYL/RCAR and type-2C protein phosphatases PP2Cs, constitute the core ABA signal module, which is responsible for the earliest ABA signaling events and participates in the regulation of plant growth, metabolism, and various stress responses [9,33,34,35,36,37,38]. SnRK2.6 controls *Arabidopsis* stomatal movement by interacting with and regulating the activity of guard cell anion channel SLAC1 [39]. SnRK2.6 also regulates seed development and dormancy by affecting the expression of extensive ABA-responsive genes [40,41]. In strawberry, FaSnRK2.6 has been found to mediate ABA-regulated fruit development and ripening mainly through the transcriptional regulation of *FaSnRK2.6* [42]. Recent studies have demonstrated that *Arabidopsis* SnRK2.6 was a key regulator in plant response to cold stress [21,25,43,44]. SnRK2.6 can phosphorylate and stabilize ICE1 protein, promoting plant freezing tolerance [25]. Recently, SnRK2.6 was found to regulate the interaction between basic transcription factor 3 (BTF3)/BTF3-like (BTF3L) protein and CBF to stabilize CBF protein, and therefore enhance *Arabidopsis* cold tolerance [44]. In contrast, *CBF* genes have been found to be less induced in tomato species in response to low temperature and cold stress, unlike *Arabidops* [45,46]. However, the roles of *Hevea brasiliensis SnRK2.6* genes in cold stress response remain unknown.

Although the molecular mechanisms of plant response to low temperature stress have been thoroughly studied in *Arabidopsis*, research on the molecular mechanism of *Hevea brasiliensis* response to low temperature stress is still in its infancy, due to the late genome sequencing and the immaturity of transgenic technology of the rubber tree. In recent years, with the completion of the rubber tree whole-genome sequencing [47,48], several key genes involved in the regulation of responses to low temperature stress in *Hevea brasiliensis* have been cloned and identified, including *HbCBF1*, *HbICE1,* and *HbICE2*, which have laid a foundation for the study of molecular mechanism underlying *Hevea brasiliensis* cold stress response [4,49,50]. However, key factors regulating the ICE/CBF signaling pathway in *Hevea brasiliensis* have not been well studied. In the present study, we investigated the effect of ABA on the cold tolerance of *Hevea brasiliensis*; identified the core ABA signaling component, SnRK2.6s, in *Hevea brasiliensis*; and evaluated their positive role in cold response. Our results provide a novel molecular mechanism underlying cold stress response in *Hevea brasiliensis*.

## 2. Results

### 2.1. ABA Promotes the Expression of Cold-Regulated Genes in the CBF Pathway in Hevea brasiliensis

**Figure 1:**
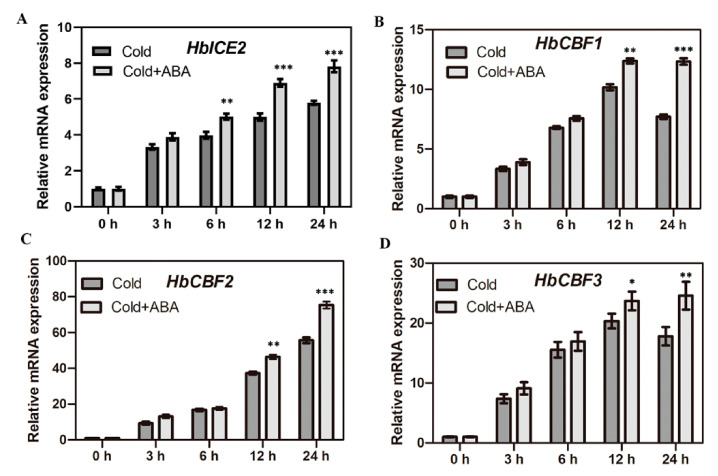
Expression of CBF pathway cold-regulated genes in response to ABA under cold stress. Leaves of rubber tree seedlings were sprayed with water or 50 µM ABA at 10 °C for a designed time. *HbeIF2* was used as internal control. Expression of: (**A**) *HbICE2;* (**B**) *HbCBF1;* (**C**) *HbCBF2;* (**D**) *HbCBF3*in rubber tree seedlings treated with cold or cold+ABA. Data are presented as mean ± SD of one experiment representative of three independent experiments. Asterisks indicate significant differences between ABA-treated seedlings and control seedlings under cold stress (*, *p* < 0.05; **, *p* < 0.01; ***, *p* < 0.001).

ABA is an important plant hormone regulating plant growth, metabolism, and various stress processes [51]. Whether ABA regulates the cold resistance of rubber trees is unknown. To explore the role of ABA in cold stress response in *Hevea brasiliensis*, first, we assayed the expression of several key cold-regulated genes in *Hevea brasiliensis* treated with ABA under cold conditions. Since ICE-CBF transcriptional cascade plays an important role in plant cold tolerance, the expression of the CBF pathway genes, including *HbICE2*, *HbCBF1*, *HbCBF2*, and *HbCBF3* of *Hevea brasiliensis* under cold stress treated with ABA for an indicated time, was tested using qRT-PCR. The results showed that the transcription level of *HbICE2* increased with the extension of low temperature treatment time and reached the highest at 24 h, while *HbICE2* transcripts in *Hevea brasiliensis* seedlings treated with both cold and ABA were higher than those in the plants treated with cold alone (Figure 1A). Similarly, the expressions of *HbCBF1–3* were significantly induced by cold stress, and ABA further enhanced their expression during the late stages of cold treatment (12–24 h) (Figure 1B–D). Collectively, these findings indicate that ABA promotes cold-regulated genes involved in the ICE/CBF transcriptional regulatory pathway in *Hevea brasiliensis*.

### 2.2. Exogenous Application of ABA Enhances Plant Cold Tolerance

**Figure 2:**
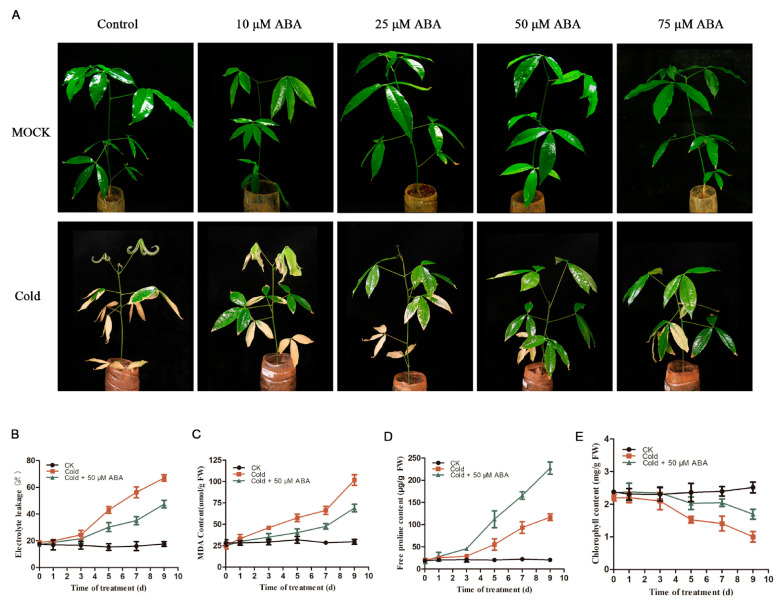
Effects of exogenous ABA on cold stress resistance in *Hevea brasiliensis*. The four-month-old *Hevea brasiliensis* seedlings grown in the soil were transferred to 10 °C and watered with water or 10 μM, 25 μM, 50 μM, or 75 μM ABA solution for the indicated days. (**A**) Phenotype of control, ABA-treated rubber tree seedlings before and after cold treatment (7 days at 10 °C). (**B**) Electrolyte leakage; (**C**) malondialdehyde (MDA) concentration; (**D**) free proline accumulation; (**E**) chlorophyll content, in CK, cold-treated (10 °C), and cold +50 μM ABA-treated leaves for the indicated time periods. CK, plants grown under normal conditions; FW, fresh weight. Error bars show standard deviation (SD) from three replicates.

Our above results showed that ABA can enhance *Hevea brasiliensis* response to cold stress by positively regulating cold-regulated genes involved in the ICE/CBF signaling pathway, prompting us to further test the role of ABA in *Hevea brasiliensis* cold stress tolerance. For this purpose, exogenous ABA was applied to the cold-treated *Hevea brasiliensis* plants, and their cold resistance phenotypes were observed. Rubber tree plants co-treated with ABA and cold had fewer wilted and necrotic leaves and exhibited fewer cold-induced injury symptoms than the plants treated with cold alone (Figure 2A). Our phenotypic analysis indicated that ABA could enhance the cold tolerance of rubber trees in a concentration-dependent manner. As compared with other tested concentrations, i.e., 50 µM and 75 µM, ABA could improve the cold tolerance of rubber trees more effectively. Therefore, a relatively low ABA concentration, i.e., 50 µM, was selected for the analysis of the cold-related physiological indicators and gene expression analysis of *Hevea brasiliensis.* In accordance with the phenotype, cold-induced cellular damage was less evident in the ABA-treated *Hevea brasiliensis* following exposure to cold stress than control plants subjected to cold temperature stress, as evidenced by significantly lower electrolyte leakage (EL) and malondialdehyde (MDA) content (Figure 2B,C). In addition, ABA-treated rubber tree leaves had higher free proline accumulation and chlorophyll levels as compared with the control plant, i.e., *Hevea brasiliensis* leaves under cold stress condition (Figure 2D,E). These results indicate that ABA positively regulates *Hevea brasiliensis* cold tolerance.

### 2.3. Identification and Bioinformatics Analysis of HbSnRK2.6s from Hevea brasiliensis

**Figure 3:**
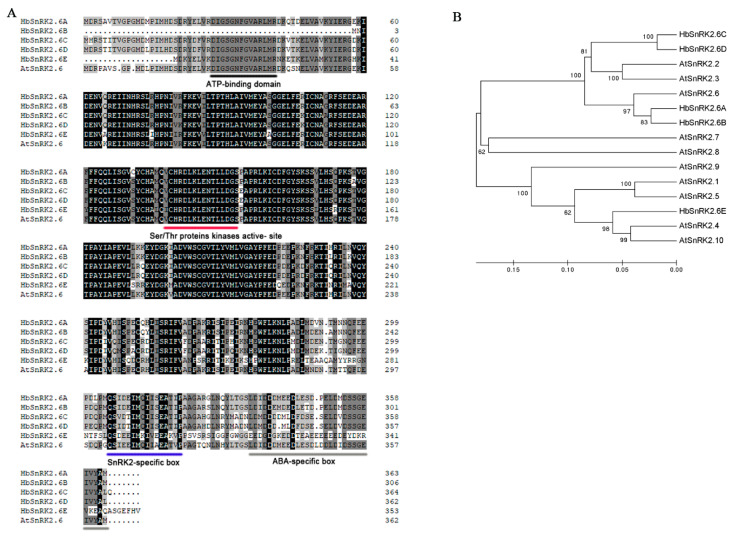
Sequence analyses of HbSnRK2.6s: (**A**) Sequence alignments of the deduced amino acid sequences of HbSnRK2.6s. Identical and similar amino acid residues indicated by black and grey shade, respectively. ATP-binding site, serine/threonine kinase protein active-site signature, SnRK2-specific box (Domain I), and ABA-specific box domain (Domain II) are labeled; (**B**) phylogenetic analysis of HbSnRK2.6 proteins with SnRK2 proteins from *Arabidopsis*.

To elucidate how ABA regulates the ICE-CBF cascade and cold tolerance in *Hevea brasiliensis*, we sought to identify the HbICE2-interacting proteins using a yeast two-hybrid screen system, since ICE-like transcription factor HbICE2 was a key positive regulator in plant cold tolerance in our previous study [49]. An ortholog of AtSnRK2.6 encoded by one CDS (designated as *HbSnRK2.6A*, accession no. MK640229) was uncovered as one of the putative HbICE2-interacting proteins. Interestingly, SnRK2.6 in *Arabidopsis* has been demonstrated to be a key factor in ABA response [25,40]. Thus, we have focused on this protein this study. *HbSnRK2.6A* and other members of the *HbSnRK2.6* family in *Hevea brasiliensis* have not been functionally characterized. A BLAST search was performed to identify HbSnRK2.6 members in the *Hevea brasiliensis* genome database using the coding sequence of *Arabidopsis* SnRK2.6 as the query sequence. Five predicted, candidate HbSnRK2.6 family genes were detected. We cloned all these *HbSnRK2.6-*like genes designated as *HbSnRK2.6A* (accession no. MK640229), *HbSnRK2.6B* (accession no. MK640230), *HbSnRK2.6C* (accession no. MK640231), *HbSnRK2.6D* (accession no. MK640232), and *HbSnRK2.6E* (accession no. MK640233). The protein sequences of the HbSnRK2.6s ranged from 306 to 364 amino acids, and the amino acid sequence alignment showed that HbSnRK2.6s and AtSnRK2.6 shared highly conserved N-terminal regions, while the C-terminus regions were divergent. In the conserved N-terminal kinase domain, all of the HbSnRK2.6 proteins contained a conserved serine/threonine kinase protein active-site signature and an ATP-binding site, except for HbSnRK2.6B which had no typical ATP-binding site (Figure 3A). In addition to the conserved kinase domain, all of the HbSnRK2.6 kinases contained two distinct domains in the C-terminal regulatory region: a SnRK2-specific box (Domain I) needed for kinase activity and an ABA-specific box domain (Domain II) required for ABA response. A phylogenetic tree was constructed using the full-length SnRK2.6 protein sequences of *Hevea brasiliensis* and SnRK2 protein sequences in *Arabidopsis*; the results showed that HbSnRK2.6A and HbSnRK2.6B were most closely related to *Arabidopsis* SnRK2.6, with the highest degree of amino acid sequence similarity with AtSnRK2.6 (Figure 3B).

### 2.4. Expression Patterns of HbSnRK2.6s in Hevea brasiliensis

**Figure 4:**
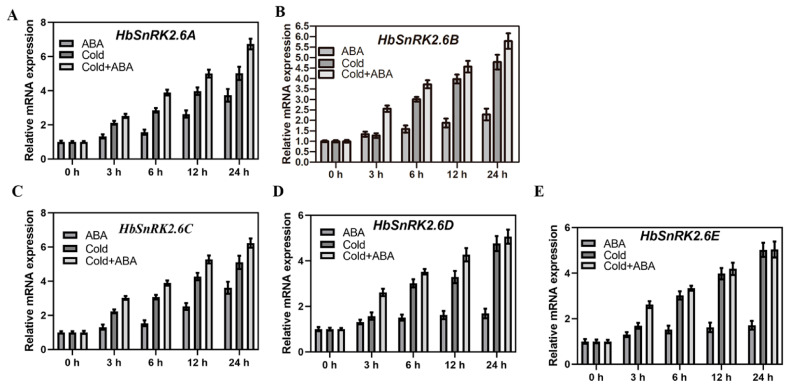
Expression profiles of *HbSnRK2.6s*. Four-month-old rubber tree seedlings were sprayed with 50 μM ABA, cold (10 °C) and cold + 50 μM ABA for the indicated time periods. *HbeIF2* was used as the reference gene. Error bars represent SD (*n* = 3). Expression of: (**A**) *HbSnPK2.6A*; (**B**) *HbSnPK2.6B*; (**C**) *HbSnPK2.6C*; (**D**) *HbSnPK2.6D*; (**E**) *HbSnPK2.6E*.

To determine the role of *HbSnRK2.6s* in *Hevea brasiliensis* response to cold stress, the expression profiles of *HbSnRK2.6s* under cold stress were also analyzed. As is shown in Figure 4, the transcript levels of *HbSnRK2.6s* were gradually increased by the cold treatment, and reached the highest levels at 24 h. The effects of ABA on the transcriptional expression of *HbSnRK2.6s* were also examined. The results showed that *HbSnRK2.6A–E* transcript levels were induced by ABA, and the transcriptional expression of *HbSnRK2.6A* and *HbSnRK2.6C* were most strongly induced by ABA. In addition, *HbSnRK2.6A*, *HbSnRK2.6B,* and *HbSnRK2.6C* transcripts in *Hevea brasiliensis* exposed to both cold and ABA treatments were significantly higher as compared with those treated with cold alone. These data suggest that *HbSnRK2.6s* are responsible for cold stress and ABA stimuli at its transcription level, and may play an important role in ABA-regulated cold tolerance in *Hevea brasiliensis*.

### 2.5. Subcellular Localization of HbSnRK2.6s

**Figure 5:**
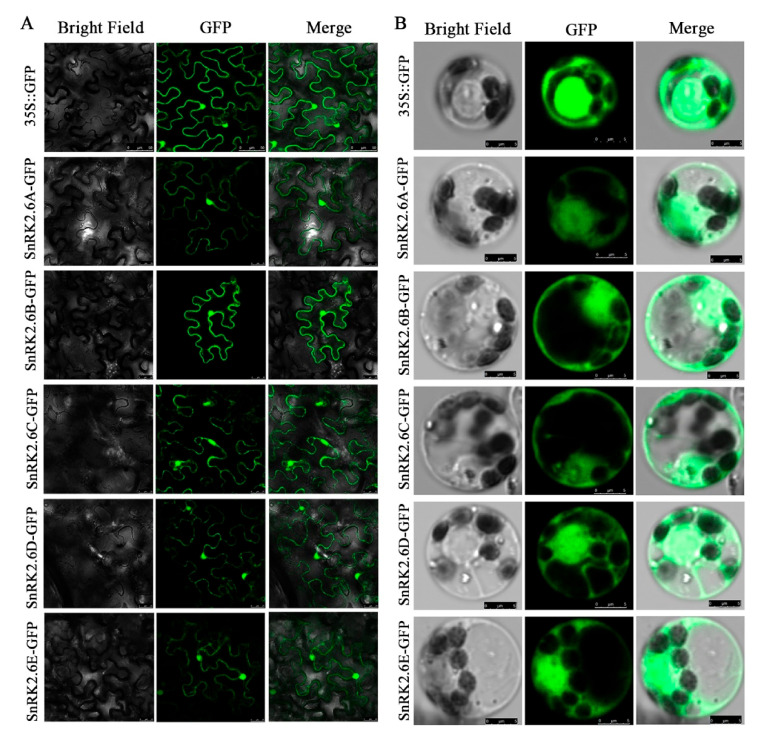
Subcellular localization of HbSnRK2.6s. GFP control vectors (35S::GFP) or 35S::HbSnRK2.6-GFP were transformed into epidermal cells of *Nicotiana benthamiana* leaves (**A**) and *Hevea brasiliensis* protoplasts (**B**). Representative images were taken under bright field and GFP fluorescence. The merged images are overlapped from two pictures on the left.

To determine the subcellular localization of HbSnRK2.6 protein, five HbSnRK2.6 proteins were fused with GFP to generate a fusion protein HbSnRK2.6s-GFP, which was driven by CaMV35S promoter. The GV3101 strain with fusion construct and empty vector was infiltrated into the leaves of *N. benthamiana* plants. The microscopic visualization showed that the control GFP fluorescence signal was distributed throughout the tobacco cells, while HbSnRK2.6s-GFP fluorescence was mainly localized in the nucleus and cytoplasm of tobacco cells (Figure 5A). To further verify the subcellular localization of the protein HbSnRK2.6s in *Hevea brasiliensis*, the subcellular localization plasmid vectors of HbSnRK2.6s were transformed into *Hevea brasiliensis* protoplasts. In accordance with the subcellular localization results in tobacco cells, all five HbSnRK2.6 proteins were mainly localized in the cytoplasm and nucleus, as well as in close proximity to the plasma membrane of rubber tree (*Hevea brasiliensis*) protoplasts (Figure 5B).

### 2.6. HbSnRK2.6A and HbSnRK2.6B Are Positive Regulators in ABA Response

**Figure 6:**
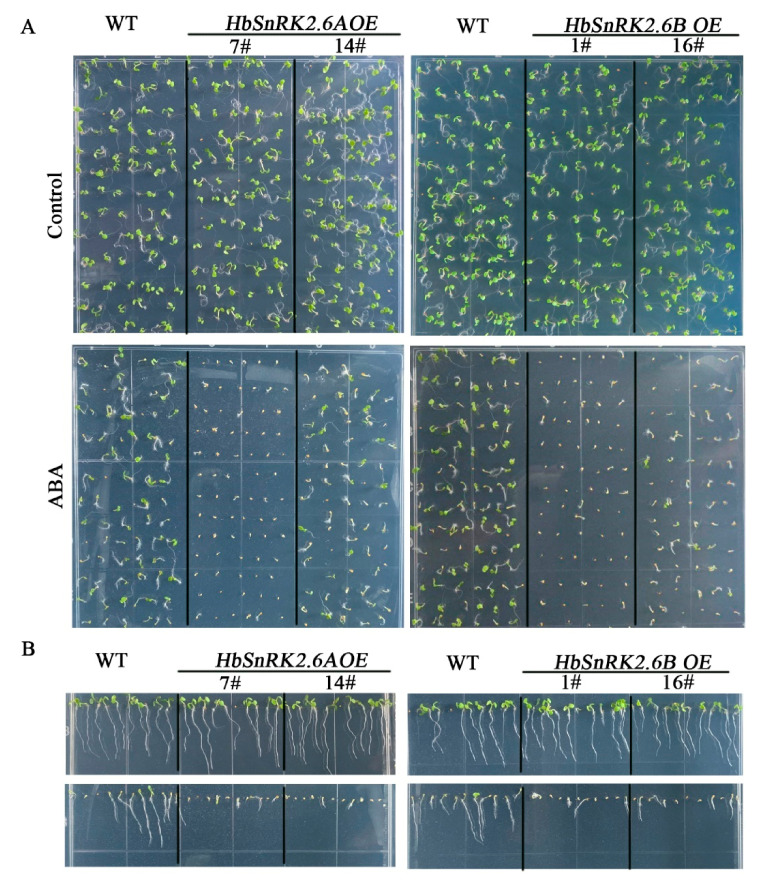
HbSnRK2.6A and HbSnRK2.6B overexpression plants are sensitive to ABA. Seed germination (**A**) and root length (**B**) of 35S::HbSnRK2.6A, 35S::HbSnRK2.6B, and WT plants on 1/2 MS supplied with or without 0.5 μM ABA.

It has been documented that SnRK2.6 proteins in other plant species are core components of ABA signaling pathway, which is involved in ABA-mediated physiological processes [40,42,52,53]. To investigate whether the HbSnRK2.6s we cloned participated in the regulation of ABA response, we overexpressed *HbSnRK2.6A* and *HbSnRK2.6B* in *Arabisopisi*s, resulting in *HbSnRK2.6A-OE* and *HbSnRK2.6B-OE* transgenic lines, and then seed germination and root growth of these overexpressing lines with or without ABA treatment were examined. We found that under normal growth conditions, the transgenic lines *HbSnRK2.6A-OE* and *HbSnRK2.6B-OE* had similar germination rates as compared with wild-type plants, whereas the seed germination rates of transgenic plants *HbSnRK2.6A-OE* and *HbSnRK2.6B-OE* were significantly lower than those of the wild-type plants when treated by ABA (Figure 6A), indicating that seed germination rates in transgenic lines *HbSnRK2.6A-OE* and *HbSnRK2.6B-OE* were relatively sensitive to ABA as compared with those of wild-type plants. Consistent with seed germination rates, the root growths of *HbSnRK2.6A-OE* and *HbSnRK2.6B-OE* lines showed hypersensitivity to ABA as compared with those of wild-type plants (Figure 6B). These results indicate that HbSnRK2.6A and HbSnRK2.6B are positive regulators of ABA response during seed germination and root growth. These combined results further confirm that HbSnRK2.6A and HbSnRK2.6B are key components of the ABA signaling pathway.

### 2.7. HbICE2 Interacts with HbSnRK2.6s

**Figure 7:**
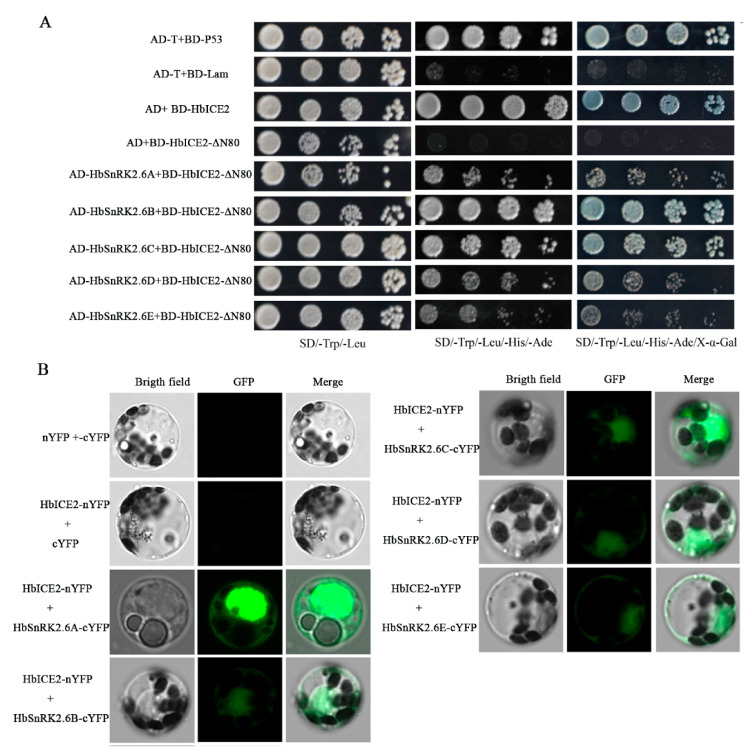
HbSnRK2.6s physically interact with HbICE2: (**A**) HbSnRK2.6s interact with HbICE2 in the yeast two-hybrid experiment; (**B**) HbSnRK2.6s interact with HbICE2 in the *Hevea* *brasiliensis* protoplasts, as indicated by the bimolecular fluorescence complementation (BiFC) assay.

To test the interaction between HbSnRK2.6A and HbICE2, a point-to-point yeast two-hybrid experiment was performed. The full-length HbSnRK2.6A and truncated HbICE2 (HbICE2-ΔN80, deletion of the transactivation region at aa 1–80) were cloned into pGADT7 and pGBKT7 vectors, respectively, generating pGADT7-HbSnRK2.6A and pGBKT7-HbICE2-ΔN80. As positive control did, yeast cells co-transformed pGADT7- HbSnRK2.6A and pGBKT7-HbICE2-ΔN80 were grown on SD/-Leu/-Trp/-His/-Ade medium. To confirm whether HbICE2 interacted specifically with HbSnRK2.6A, other HbSnRK2.6s including HbSnRK2.6B, HbSnRK2.6C, HbSnRK2.6D, and HbSnRK2.6E were cloned and analyzed. As shown in Figure 7A, HbICE2 interacted with all five HbSnRK2.6 proteins. In addition, a BiFC assay was conducted in *Hevea* *brasiliensis* protoplasts to verify the interactions in vivo. Co-expressing HbICE2-nYFP and HbSnRK2.6s-cYFP could generate florescence signal in *Hevea* *brasiliensis* protoplasts (Figure 7B), implicating that HbICE2 could interact with HbSnRK2.6 in vivo. The yeast two-hybrid and BiFC assays both collectively indicated that HbICE2 could physically interact with HbSnRK2.6s.

### 2.8. Overexpression of HbSnRK2.6A and HbSnRK2.6B in Arabidopsis Enhances Cold Stress Tolerance

**Figure 8:**
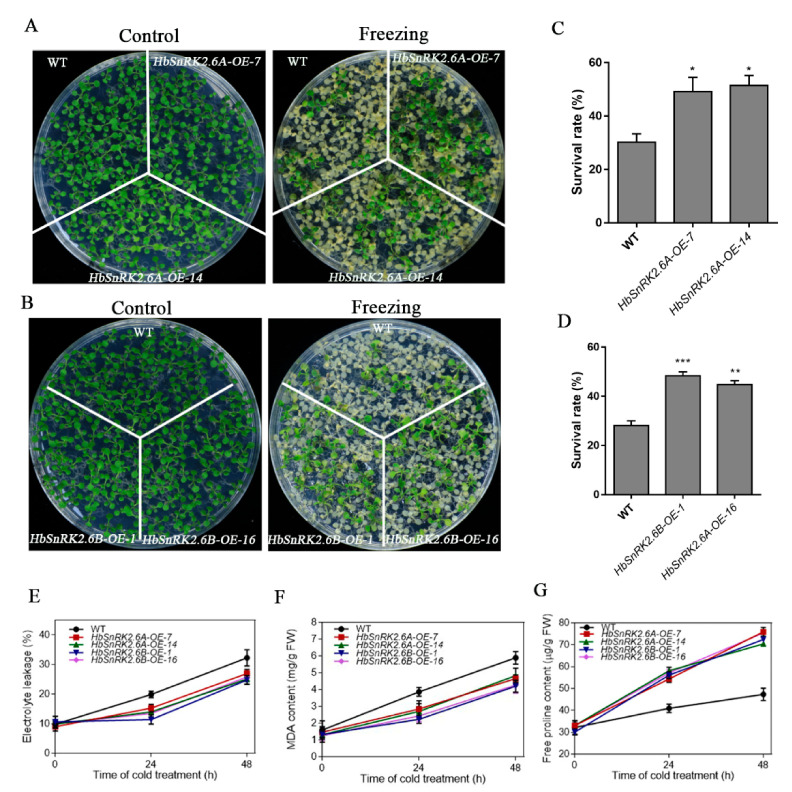
Overexpression of *HbSnRK2.6A* and *HbSnRK2.6B* confers enhanced cold tolerance in *Arabidopsis*. Two-week-old seedlings were pretreated at −4 °C for 2 days, followed by −8 °C for 4 h, and finally recovered under normal conditions for 7 days, freezing phenotypes (**A**,**B**) and survival rates (**C**,**D**) of transgenic and WT plants were analyzed after 7 days recovery. Electrolyte leakage (**E**), malondialdehyde (MDA) concentration (**F**), and free proline content (**G**) in transgenic and WT plants before and after cold treatment for time indicated. Data shown are mean ±SD of three independent experiments, and asterisks indicate significant differences at * *p* < 0.05, ** *p* < 0.01, and *** *p* < 0.001 (Student’s *t*-test).

Given that our experiments have demonstrated that HbSnRK2.6A/B could physically interact with HbICE2, a crucial positive regulator in a cold signaling pathway, efforts were then made to explore the role of HbSnRK2.6A/B in the tolerance to cold stress. The *Arabidopsis* transgenic lines overexpressing *HbSnRK2.6A* (*HbSnRK2.6A-OE-7* and *HbSnRK2.6A-OE-14*) and *HbSnRK2.6B* (*HbSnRK2.6B-OE-1* and *HbSnRK2.6A-OE-16*) were used for cold stress assays. Under normal growth conditions, there was no significant differences in morphology and survival rates between *HbSnRK2.6A/B* overexpressing transgenic lines and wild-type plants. When exposed to cold stress, transgenic lines were more resistant to freezing treatment as compared with wild-type plants (Figure 8A,B), and the survival rates of *HbSnRK2.6A/B* overexpressing transgenic lines were significantly higher than those of wild-type seedlings after freezing temperatures treatment (Figure 8C,D), indicating that overexpression of *HbSnRK2.6A/B* could enhance tolerance of the *Arabidopsis* plants to cold stress. Several physiological parameters such as electrolyte leakage (EL), malondialdehyde (MDA) level, and proline content are known indicators of cell injuries caused by the stresses. To further confirm the role of *HbSnRK2.6A/B* on cold tolerance, the above physiological parameters were measured. The EL and MDA contents in these transgenic plants were both consistently lower as compared with those in the wild-type plants when exposed to cold stress (Figure 8E,F), indicating that transgenic lines suffered less cold-induced cellular damage than wild-type seedlings. In addition, when subjected to cold treatment, the proline levels in the transgenic lines were significantly higher than those in WT plants (Figure 8G). Taken together, these findings suggest that *HbSnRK2.6A/B* contribute positively to cold tolerance.

### 2.9. HbSnRK2.6A/B Positively Regulate the Expression of Cold-Responsive Genes

**Figure 9:**
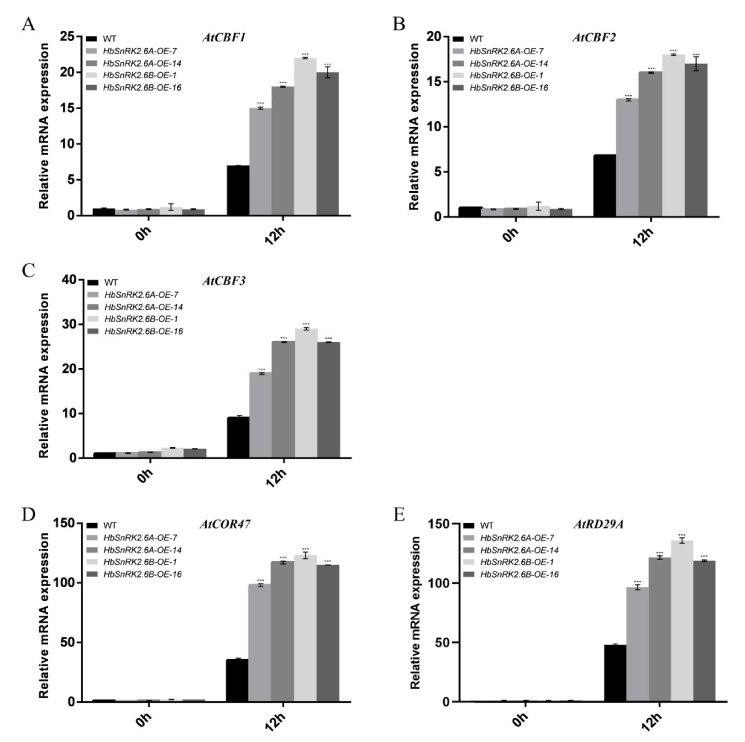
Expression of *CBFs* and their regulons in *HbSnRK2.6A* and *HbSnRK2.6B* overexpression lines treated by cold stress. Two-week-old plants grown in soil were exposed to 4 °C for the time indicated. Expression of: (**A**) *AtCBF1*; (**B**) *AtCBF2*; (**C**) *AtCBF3*; (**D**) *COR47*; (**E**) *RD29A*, in transgenic and WT plants were analyaed. Data are presented as mean ± SD of one experiment representative of three independent experiments,and asterisks indicate significant differences at *** *p* < 0.001 (Student’s *t*-test).

To reveal whether *HbSnRK2.6A/B*-regulated plant cold stress responses are dependent on the CBF signaling pathway, the transcript levels of *Arabidopsis* CBF cold-responsive pathway genes, including *AtCBF1–3*, *AtCOR47*, and *RD29A*, were analyzed by qRT-PCR. As is shown in Figure 9, the expressions of *AtCBF1–3*, *AtCOR47,* and *RD29A* were dramatically induced by cold in both *HbSnRK2.6A-OE* and *HbSnRK2.6B-OE* lines as compared with those in wild-type plants, while this induction was much stronger in the overexpressing lines than that in the wild type. These results imply that *HbSnRK2.6A/B* positively regulate the expression of CBF cold-responsive pathway genes under cold conditions, thereby, contributing to enhanced cold resistance.

### 2.10. HbSnRK2.6A/B Promote HbICE2 Transcriptional Activity

**Figure 10:**
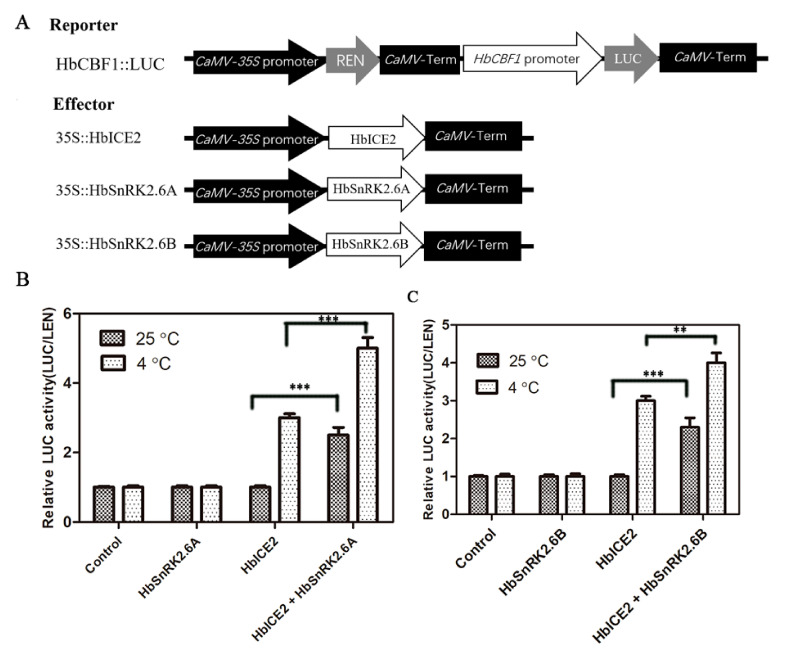
HbSnRK2.6A and HbSnRK2.6B are direct transcriptional activators of HbICE2: (**A**) Schematic diagrams of effector and reporter constructs for dual-LUC transient expression assay; (**B**,**C**) relative LUC activity of *HbCBF1* promoter in tobacco leaves with transient expression of *35S::HbICE2*, *35S::HbICE2* + *35S::HbSnRK2.6s*. The *Agrobacterium tumefaciens* GV3101 harboring *HbCBF1::LUC* and other effector plasmids were transferred into tobacco leaves, leaves transfected with *HbCBF1::LUC* only were used as a control. After infiltration for 48 h, the infected tobacco plants were exposed to 4 °C for 4 h, and then relative LUC activity of *HbCBF1* promoter was quantified by measuring relative LUC and REN activities. Data shown are mean ± SD of three independent experiments, and asterisks indicate significant differences at ** *p* < 0.01 and *** *p* < 0.001 (Student’s *t*-test).

Since HbSnRK2.6A/B could interact with HbICE2, it is possible that the HbSnRK2.6A/B–HbICE2 interaction may interfere with the transcriptional activity of transcription factor HbICE2. To verify this assumption, transient transactivation assays were performed using firefly LUC gene driven by *HbCBF1* promoter as a reporter. HbSnRK2.6A/B and HbICE2 effector plasmids were generated by fusing the constitutive CaMV35S promoter with the HbSnRK2.6A/B and HbICE2 (Figure 10A). When reporter construct HbCBF1::LUC was transformed together with the effector plasmid 35S::HbICE2 into leaves of *N. benthamiana*., the LUC activity was significantly increased as compared with infiltration of HbCBF::LUC alone, suggesting that HbICE2 transactivated the expression of *HbCBF1*. Moreover, under cold treatment, co-expression of reporter plasmid *HbCBF1::LUC* with effector plasmids *35S::HbSnRK2.6A* or *35S::HbSnRK2.6A* and *35S::HbICE2* generated much higher LUC activity than infiltration of *HbCBF1::LUC* alone or co-expression of *HbCBF1::LUC* with *35S::HbICE2* (Figure 10B), indicating that HbSnRK2.6A/B promote the HbICE2-induced *HbCBF1* expression. These results suggest that HbSnRK2.6A/B enhance cold tolerance by promoting the transcriptional ability of HbICE2.

## 3. Discussion

*Hevea brasiliensis* is a typical tropical rainforest tree species, and therefore low temperature significantly suppresses its growth and development and also restricts its geographical distribution and latex production [3,4]. Hence, improvement of cold tolerance in rubber tree varieties is of great importance. However, the molecular mechanisms of rubber tree response to cold stress are still largely unknown. In the present study, we investigated the role and molecular mechanism of ABA in *Hevea brasiliensis* cold stress responses. We found that ABA can increase cold induction of *HbSnRK2.6A, HbSnRK2.6B,* and *HbSnRK2.6C* transcripts and significantly enhance *Hevea brasiliensis* cold tolerance. In addition, overexpression of *HbSnRK2.6A/B* increased plant cold resistance. Furthermore, HbSnRK2.6s can physically interact with HbICE2 and promote its transcriptional activity, increasing *HbCBF1* expression, thereby, rendering an enhanced cold tolerance of *Hevea brasiliensis.* Interestingly, in rubber trees, ABA is an important hormone that can stimulate latex synthesis, and exogenous application of ABA significantly increased latex yield by 4.3 times [54,55]. Although whether and how HbSnRK2.6, the key player in ABA signaling, functions in latex production are still unknown, our study on the mechanism of ABA-HbSnRK2.6 module regulating the cold tolerance of rubber trees is helpful to the breeding of rubber tree varieties with both increased cold resistance and high latex yield.

SnRK2s are central components in ABA-triggered signaling pathway [9,31,51,52]. In our study, *HbSnRK2.6s* (*HbSnRK2.6A–E*) in *Hevea brasiliensis* were cloned and functionally characterized. Amino acid sequence alignment showed that HbSnRK2.6s share high sequence similarity with AtSnRK2.6, and all HbSnRK2.6s have typical structural signatures of SnRK2 family proteins, including serine/threonine kinase protein active-site signature, ATP-binding site, SnRK2-specific box (Domain I), and ABA-specific box domain (Domain II); however, HbSnRK2.6B has no typical ATP-binding site (Figure 3). Consistent with SnRK2 members in many other plant species, HbSnRK2.6s showed highly conserved N-terminal domains but divergent C-terminus regions (Figure 3) [56,57,58,59]. The conserved domain of HbSnRK2.6B was lost, suggesting that subfunctionalization may have occurred during the long-term evolution of rubber tree. This phenomenon has also been observed in PbrSnRK2 proteins in pear [59].

Previous studies have demonstrated that exogenous application of ABA could enhance cold tolerance in some plants and may be correlated with cold-induced physiological alteration [39,60,61,62,63]. SnRK2.6 function is necessary in plant cold stress tolerance as *snrk2.6 Arabidopsis* mutant is very sensitive to cold stress, while *HbSnRK2.6*-overexpressing transgenic *Arabidopsis* plants display enhanced cold stress tolerance (Figure 2) [25]. However, cold-activated kinase activity of SnRK2.6 is independent of ABA in *Arabidopsis* [64], while our study showed that exogenous ABA treatment extensively upregulated the expression of *HbICE2*, *HbCBF1*, *HbCBF2,* and *HbCBF3* in cold-treated rubber seedings, resulting in increased cold stress tolerance of rubber seedlings, implying a distinct role of ABA in cold response of rubber trees and *Arabidopsis*. We speculated that the different effects of ABA on cold responses in rubber trees and *Arabidopsis* plants may be dependent on plant species.

Our study showed that *HbSnRK2.6A-* or *HbSnRK2.6B-*overexpressing *Arabidopsis* plants are hypersensitive to ABA in terms of seed germination and root elongation as compared with the wild-type *Arabidopsis* plants (Figure 6), revealing that HbSnRK2.6 plays a conserved role with its homologous protein AtSnRK2.6 in *Arabidopsis* in ABA signaling transduction. SnRK2.6 usually plays its role in ABA-mediated stress responses through phosphorylating downstream factors [9,39]. Indeed, several crucial phosphorylation substrates of AtSnRK2.6/OST1 have been identified in plant cold stress responses including AtICE1, BTF3, and BTF3-like protein, which elicits different effects on promoting plant cold stress tolerance [44]. In our study, we found that both HbSnRK2.6A and HbSnRK2.6B could significantly enhance the transcription activity of HbICE2 on the expression of *HbCBF1*, suggesting that HbSnRK2.6 may also phosphorylate HbICE2, but additional analysis is needed to draw a firm conclusion.

MDA is a lipid peroxidation marker, which is produced and accumulated in various abiotic stresses including cold stresses [10,50]. We have also noticed that cold stress-induced MDA accumulation in the wild-type plant could be significantly repressed by the overexpression of *HbSnRK2.6A* or *HbSnRK2.6B*, suggesting another role of HbSnRK2.6 in plant cold stress tolerance through regulating the expression of reactive oxygen species (ROS)-metabolizing enzymes directly or indirectly. Recently, a large number of *AtCBFs* downstream target genes were identified in *Arabidopsis* [65], shedding a light on whether and how HbSnRK2.6-ICE2 module functions in the maintenance of ROS homeostasis by targeting the genes encoding ROS-scavenging enzymes.

Stomata are the specialized epidermal cells in leaf, and control both the water transpiration and gas exchange in plants [66]. In addition to the activation of the ICE/CBF signaling pathway during plant cold response, SnRK2.6/OST1 was initially identified as an important central player that could extensively promote stomatal closure under drought or dehydration stress conditions by phosphorylating membrane ion channel proteins such as slow anion channel-associated 1 (SLAC1) [67]. As stomatal closure restricts leaf water transpiration, and thus decreases the loss of leaf heat, we still cannot exclude the possibility that ABA- and HbSnRK2.6-conferred cold stress tolerance in rubber tree seedlings is at least partially due to higher plant foliar temperature caused by smaller stomatal aperture and slower water transpiration.

In addition to the stimulation of kinase activity of OST1/SnRK2.6, cold also significantly increases the mRNA abundance of *HbSnRK2.6s* in rubber tree (Figure 4), consistent with previous results that transcripts of *SnRK2s* are upregulated under cold stress in other plants including maize, wheat, and *Arabidopsis* [41,62,64], suggesting a conserved mechanism underlying the regulation of *SnRK2.6* expression across different plant species. We also found that ABA could increase cold induction of *HbSnRK2.6A*, *HbSnRK2.6B,* and *HbSnRK2.6C* transcripts. These results, in combination with our other findings that *HbSnRK2.6s* could enhance plant cold tolerance, and HbSnRK2.6 could promote the transcriptional activity of HbICE2 by interacting with HbICE2 to induce *HbCBF1* expression, strongly suggest that the transcriptional regulation of *HbSnRK2.6s* is involved in *HbSnRK2.6s*-mediated ABA-regulated *Hevea brasiliensis* cold tolerance. These data are also consistent with previous results in strawberry fruit that *FaSnRK2.6* mediates ABA-regulated fruit ripening mainly through the transcriptional regulation of *FaSnRK2.6* [42].

## 4. Materials and Methods

### 4.1. Plant Materials and Growth Conditions

The *Hevea* *brasiliensis* cultivars ReYan 7-33-97 seedlings were purchased from CATAS (Chinese Academy of Tropical Agricultural Sciences) and cultivated in pots in the greenhouse of Hainan University. In order to analyze the effect of ABA on *Hevea* *brasiliensis* cold tolerance, four-month-old *Hevea brasiliensis* seedlings were watered in the soil with water or ABA solution, and then transferred to plant growth chamber set at 10 °C. Phenotypic observation was carried at 7 days after treatment. To investigate the effect of ABA on the expression of *HbSnRK2.6s* and cold-responsive genes, ABA was sprayed onto the leaves of rubber tree seedlings. Leaves were collected at 0, 3, 6, 12, and 24 h after ABA treatment for RNA extraction.

*Arabidopsis* ecotype Columbia (Col) plants and overexpressing *HbSnRK2.6A/B* transgenic *Arabidopsis* lines were grown in a greenhouse at 23 °C under a 16:8 h light/ dark photoperiod. To generate the transgenic plants, the plants’ overexpression vectors, pBWA(v) harboring full-length cDNA of *HbSnRK2.6A* or *HbSnRK2.6B,* were transferred into wild-type *Arabidopsis* by an *A. tumefaciens* (GV3101)-mediated transformation and the floral dip method [68]. Transgenic lines were selected on 1/2 MS medium supplied with 30 μg mL^−1^ Basta resistance, and positive transgenic lines were confirmed by genomic PCR and RT-qPCR analyses. Primers used are listed in Supplementary Table S1. T4 transgenic plants were used for further analysis. For *Arabidopsis* cold tolerance, assays were performed as described by Yuan et al. [50].

### 4.2. Physiological Measurements

For physiological analyses, 4-month-old *Hevea brasiliensis* seedlings treated with 0 µM ABA or 50 µM ABA were placed in a growth chamber at 10 °C for the designated time points, and 14-day-old transgenic *HbSnRK2.6A/B* and WT *Arabidopsis* grown in nutrient soil were kept in a controlled-environment growth chamber at 4 °C for 0, 24, or 48 h. After treatment, leaves were collected, and then electrolyte leakage and proline accumulation were quantified, according to Yuan et al. [50]. Briefly, for electrolyte leakage measurement, the harvested leaves were placed in a bottle containing 40 mL deionized distilled water, and the bottle was shaken on a shaker at 120 rpm for 3 h at room temperature. The initial conductivity of sample (C1) was measured by a conductivity meter. After boiling the leaves for 30 min and shaking the bottle for 1 h at room temperature, the conductivity (C2) was measured. Electrolyte leakage was calculated using the following equation: C (%) = (C1/C2) × 100%. For proline content measurement, the collected leaves were weighed, and then extracted in 3% sulfosalicylic acid. The supernatant (2 mL) was mixed with glacial acetic acid (2 mL) and ninhydrin reagent (2 mL). After boiling the reaction mixture for 40 min, the reaction was terminated in an ice bath. The reaction mixture was extracted with toluene (5 mL) and the proline content was calculated based on the absorbance at 520 nm. MDA contents were measured, as previous described [69]. Briefly, MDA was extracted with thiobarbituric acid (TBA), and MDA content was calculated based on the absorbance at 450, 532, and 600 nm. Chlorophyll level was measured according to previous description [70].

### 4.3. RNA Extraction and qPCR Analysis

Total RNA from *Hevea brasiliensis* and *Arabidopsis* was extracted, according to manufacturer’s instructions (BioTeke, Beijing, China). First-strand complementary cDNA was synthesized using a commercial kit (K1622, Thermo Fischer, MA, USA). Quantitative real-time PCR (qRT-PCR) was performed, according to a previous description [50]. Briefly, qRT-PCR was carried out on an ABI-7500 real-time PCR system with SYBR green PCR Mix (TaKaRa, Tokyo, Japan). The genes *HbeIF2* and *AtEIF4* were chosen as reference genes for *Hevea brasiliensis* and *Arabidopsis*, respectively. The relative expression levels were normalized to reference genes through 2^−∆∆CT^ method. Three independent biological replicates were used for data analysis, and each biological sample contained three technical replicates. The primers are listed in Supplementary Table S1.

### 4.4. Bioinformatics Analysis

To identify SnRK2.6s from *Hevea brasiliensis*, a BLAST was performed in the *Hevea brasiliensis* genome database using the coding sequence of *Arabidopsis* SnRK2.6 as the query sequence. Five sucrose nonfermenting1-related protein kinases were identified, which designated as *SnRK2.6A–E*. Sequence alignment was generated using the DNAMAN program. A neighbor-joining analysis was carried out to construct a phylogenetic tree by software MEGA 6.0.

### 4.5. Yeast Two-Hybrid Assays

The full-length cDNA sequences of HbSnRK2.6s were fused with GAL4 activation domain in the pGADT7 vector to get recombinant vectors, pGADT7-HbSnRK2.6s; the truncated CDS of HbICE2 (deletion of the N-terminal transactivation region at aa 1–80) was fused in frame downstream of the binding domain of pGBKT7 to generate pGBKT7-HbICE2-ΔN80. The primers are listed in Supplementary Table S1. The recombinant bait and prey constructs were co-transformed into AH109 yeast strain by the LiAc method, according to the manufacturer’s instructions (Clontech, San Francisco, CA, USA). Transformed colonies were screened on selective SD medium at 28 °C to test for possible interactions.

### 4.6. BiFC Assays

To generate the BiFC recombinant constructs, the ORFs without a stop codon of HbSnRK2.6s and HbICE2 were inserted into pSPYNE-35S and pSPYCE-35S vectors, respectively, and confirmed by sequencing. Primers are listed in Supplementary Table S1. The recombinant plasmids were transformed into *Agrobacterium tumefaciens* GV3101 strain. Rubber tree mesophyll cell protoplasts were extracted and transformed, as previously described [71]; transformed protoplasts were incubated for 12 h in low light situations, and then YFP fluorescence was detected by a Leica TCS SP8 confocal laser scanning microscope.

### 4.7. Subcellular Location

The cDNA sequences of *HbSnRK2.6s* were cloned into the pBWA(v)BS vector. For subcellular localization of HbSnRK2.6s in tobacco leaf epidermal cells, the *Agrobacterium tumefaciens* GV3101 strain containing recombinant constructs were infiltrated into *N. benthamiana* leaf, according to a previous study [72]. Two days after infiltration, HbSnRK2.6 subcellular locations were detected by visualizing GFP fluorescence under a Leica TCS S98 confocal laser scanning microscope. For subcellular localization of HbSnRK2.6s in rubber tree protoplasts, rubber tree mesophyll cell protoplast extraction and transformation were performed, as previously described [71]. After transformation for 12 h, GFP fluorescence was examined using confocal laser scanning microscope (Leica TCS SP8).

### 4.8. Transient Transactivation Assay

HbCBF1promoter was cloned to pGreenII0800-LUC to generate reporter construction 35S::REN-pHbCBF1::LUC, the full-length CDSs of HbICE2 and HbSnRK2.6s were under control of CaMV35S promoter to form effector plasmids pGreenII62-SK-HbICE2 and pGreenII62-SK-HbSnRK2.6s. The plasmids were introduced into *Agrobacterium tumefaciens* GV3101 strain. The transient expression was carried out in tobacco (*N. benthamiana)* leaves, based on a previous method [72]. The infected tobacco plants were grown at 25 °C for 2 days, and then exposed to 4 °C for 4 h. Transient expression was quantified by measuring relative LUC and REN activities using the Dual Luciferase Reporter Gene Assay Kit (RG027, Beyotime, Shanghai, China), according to the manufacturer’s instructions.

### 4.9. Statistical Analysis

All experiments were performed in three independent repetitions. All data are shown as means ± standard deviation (SD), which were statistically analyzed by ANOVA or by Student’s *t*-test using GraphPad PRISM v.8.0 software. Different asterisks indicate significant differences at *** *p* < 0.001, ** *p* < 0.01, and * *p* < 0.05.

## Data Availability

Not applicable.

## Supplementary Materials

The following are available online at https://www.mdpi.com/article/10.3390/ijms222312707/s1.
