# Supplementary material for: HbSnRK2.6 Functions in ABA-Regulated Cold Stress Response by Promoting HbICE2 Transcriptional Activity in Hevea brasiliensis
Source: Int J Mol Sci. 2021 Nov 24;22(23):12707. doi: 10.3390/ijms222312707 (PMC8657574; doi:10.3390/ijms222312707)
Supplement: Supplementary file 1 [file ijms-22-12707-s001.zip › ijms-1449259-supplementary.pdf]

Supporting Information

Table S1 List of the Primers Used in this study.

Primer name	Sequence (5' to 3')
AD-SnRK2.6A-F	GGGCATCGATACGGGATGGATCGTTCGGCGGTTACCGTA
AD-SnRK2.6A-R	AGCTCGAGCTCGATG TCACATTGCATATACTATCTCTCCAC
AD-SnRK2.6B-F	GGGCATCGATACGGGATGAACATAGATGAAAATGTACAAAGGG
AD-SnRK2.6B-R	AGCTCGAGCTCGATGCATGGCATATACTATCTCTCCACTAC
AD-SnRK2.6C-F	GGGCATCGATACGGGATGCATCGATCGACGATTACTGTGG
AD-SnRK2.6C-R	AGCTCGAGCTCGATGTTATTGCAATGCATAAACAATCTCCC
AD-SnRK2.6D-F	GGGCATCGATACGGGATGGATCGGTCGACGATAACCGTG
AD-SnRK2.6D-R	AGCTCGAGCTCGATGTTACAGTGCATAAACTATCTCCC
AD-SnRK2.6E-F	GGGCATCGATACGGGATGGACAAGTACGAGCTTGTGAAGG
AD-SnRK2.6E-R	AGCTCGAGCTCGATGTTAACTCACATGAAATTCTCCACTTGC
YCE- SnRK2.6A-F	cgcgccactagtggaATGGATCGTTCGGCGGTT
YCE- SnRK2.6A-R	gacagtactatcgatCATTGCATATACTATCTCTCC
YCE- SnRK2.6B-F	cgcgccactagtggaATGAACATAGATGAAAAT
YCE- SnRK2.6B-R	gacagtactatcgatCATGGCATATACTATCTC
YCE-SnRK2.6C-F	cgcgccactagtggaATGCATCGATCGACGATT
YCE-SnRK2.6C-R	gacagtactatcgatTTGCAATGCATAAACAAT
YCE-SnRK2.6D-F	cgcgccactagtggaATGGATCGGTCGACGATA
YCE-SnRK2.6D-R	gacagtactatcgatCAGTGCATAAACTATCTC
YCE-SnRK2.6E-F	cgcgccactagtggaATGGACAAGTACGAGCTT
YCE-SnRK2.6E-R	gacagtactatcgatACTCACATGAAATTCTCC
SnRK2.6AOE-F	ATGGATCGTTCGGCGGTTACCGTA
SnRK2.6AOE-R	TCACATTGCATATACTATCTCTCCAC
SnRK2.6BOE-F	ATGAACATAGATGAAAATGTACAAAGGG
SnRK2.6BOE-R	CATGGCATATACTATCTCTCCACTAC
qRT-SnRK2.6A-F	TTACCGTAGGTCCCGGTATG
qRT-SnRK2.6A-R	ATGCCTCAATGACCTGTGGT
qRT-SnRK2.6B-F	TGGAGTATGCATCTGGTGGA
qRT-SnRK2.6B-R	TGTGAATGGAGAACCGATGA
qRT-SnRK2.6C-F	GATTACTGTGGGGCCTGGTA
qRT-SnRK2.6C-R	GCCTGAGGGACCTGTGATTA

**qRT-SnRK2.6D-F**	TATGGATTTGCCGATCTTGC
**qRT-SnRK2.6D-R**	ACAATGTTGGGATGCCTGAG
**qRT-SnRK2.6E-F**	AGGCTCATGAGGAACAAGGA
**qRT-SnRK2.6E-R**	TTGCAGAGCATGGCAGTAAC
**qRT-HbICE2-F**	GCTCTCTGGACTAAATGGCAGT
**qRT-HbICE2-R**	TCCTTGCTCTCCATCACATCAC
**qRT-HbCBF2-F**	GCATCTCCAGCCTAGGATAA
**qRT-HbCBF2-R**	GCCCTCAATTCAACCTTTCA
**qRT-HbCBF1-F**	CAATGCCAGCTTCTTCATAAAC
**qRT-HbCBF1-R**	CGTTCCAATTCAACCTCTCC
**qRT-HbeIF2-F**	CGACCTTTGATCCGTTTGCT
**qRT-HbeIF2-R**	CTTCCTACCATTCCGTTGCT
**qRT-AtCBF1-F**	TGAAGTGAGAGAGCCAAACAAGA
**qRT-AtCBF1-R**	CCGAGTCAGCGAAGTTGAGA
**qRT-AtCBF2-F**	CTCACGACGTCGCCGCCATA
**qRT-AtCBF2-R**	GCTTCAGCCGCCGCCTTTTG
**qRT-AtCBF3-F**	GGCGGAACAGAGCGAAAA
**qRT-AtCBF3-R**	GAAGCGGCAAAAGCATC
**AtCOR47qRT-F**	GGCTGAGGAGTACAAGAACAA
**AtCOR47qRT-R**	ACAATCCACGATCCGTAACC
**AtRD29AqRT-F**	GCTTTCTGGAACAGAGGATGTA
**AtRD29AqRT-R**	CGACTCTTCCTCCAACGTTATC
**AtPDF2qRT-F**	TAACGTGGCCAAAATGATGC
**AtPDF2qRT-R**	GTTCTCCACAACCGCTTGGT
**AtEIF4qRT-F**	GCACAGTTTGATGATGCACGTCAGT
**AtEIF4qRT-R**	GGTTCTCTTGAAGACCCATGGCA
